# Multi-level clustering and Prediction based energy efficient routing protocol to eliminate Hotspot problem in Wireless Sensor Networks.

**DOI:** 10.1038/s41598-024-84596-6

**Published:** 2025-01-07

**Authors:** Bhaskar Prince, Prabhat Kumar, Sunil Kumar Singh

**Affiliations:** 1 Department of Computer Science and Engineering, National Institute of Technology Patna, Patna, Bihar India; 2 Department of Computer Science & Engineering, Manipal Institute of Technology Bengaluru, Manipal Academy of Higher Education, Manipal, Karnataka India

**Keywords:** Mega-Cluster Head (MCH), Cluster-Head (CH), Wireless Sensor Networks (WSN), Data Mule, Computer science, Information technology, Information theory and computation, Electrical and electronic engineering

## Abstract

Conserving energy of sensor nodes and ensuring balanced workloads among them are fundamental concerns in Wireless Sensor Network (WSN) design. Clustering strategies offer a promising avenue to minimize node energy consumption, thereby prolonging network lifespan. Nevertheless, numerous multi-hop routing protocols using clustering technique face the challenge of nodes nearer to the Base Station (BS) depleting their energy faster due to forwarding data from the entire network leading to premature node failure and network partitioning known as ‘hotspot problem’. The paper introduces an Energy-Efficient Mega-Cluster-Based Routing (EEMCR) protocol specially designed for expansive coverage area. The primary principle behind designing this protocol is to eliminate the hotspot problem and restrict the transmission range of nodes to the threshold distance defined by the radio energy model, thereby enhancing the overall network lifespan. The protocol adopts a centralized approach employing fixed clustering wherein the BS partitions the network into square-shaped clusters. The cluster size is determined by the threshold transmission range of the sensor radio energy model, guaranteeing that all network communication stays within this threshold distance. Four such clusters form a mega-cluster with a Mega-Cluster-Head (MCH) elected among the four Cluster Heads (CHs). The MCH role is evenly distributed among nodes of all four clusters in subsequent rounds for uniform distribution of its overhead. Implementing data aggregation at two levels (CH level as well as MCH level) leads to reduced data traffic and energy consumption throughout the network. Moreover, data collection by two data mules based on odd–even round number ensures balanced data traffic and energy distribution across the network. Analysis indicates that the proposed protocol effectively mitigates the hot-spot problem and reduces data transmission overhead of sensor nodes. In simulation, the proposed protocol on an average improves network life by 34.5%, 23.5%, 14.5% and 5.5% as compared to existing protocols FCEEC, DBSCAN, LPGCR and FBECS respectively for deployment of nodes between 600 to 1200. Also, approximately 46%, 32%, 21% and 14% of lesser sensor nodes are dead for proposed protocol in respective rounds as compared to existing protocols FCEEC, DBSCAN, LPGCR and FBECS respectively. Comparative evaluations demonstrate improved network lifetime when compared to equivalent recent routing protocols.

## Introduction

Wireless Sensor Networks (WSNs)^1^ are composed of tiny sensor nodes deployed strategically to observe surrounding parameters such as temperature, humidity and pressure^2^. Typically, a stationary base station (BS) serves as the central communication hub for all sensor nodes, positioned either within or outside the network. In extensive networks, sensor nodes frequently utilize multi-hop routing to relay data to the stationary BS, as energy consumption escalates with distance from the BS. These routing scheme not only conserves energy but also enhances protocol efficiency. However, despite its advantages, multi-hop routing protocols face a significant challenge recognized as the hot-spot problem^3^. In multi-hop routing, distant nodes from the BS transmit data through one-hop range neighbors known as relay nodes. These relay nodes then forward aggregated data to upper-layer relay nodes along the path to the BS. Nodes near the BS often experience higher data transmission demands resulting in premature energy depletion and the appearance of a hot-spot in close proximity of the BS. Hence, nodes possessing sufficient energy also encounter difficulties in transmitting data to the BS. Researchers commonly tackle this issue using three methods: (i) Clustering (ii) heterogeneity and (iii) mobile elements. Clustering is widely acknowledged as an efficient routing strategy in WSNs in which clusters are formed by grouping the nodes. Each cluster is supervised by a coordinator node known as CH which are selected based on specific parameters^4^. CHs gather and consolidate data from member nodes before relaying it to relay nodes or the BS. However, CH nodes often experience faster energy depletion compared to other members due to additional overhead. To address this imbalance, the CH role is rotated among member nodes to evenly distribute energy consumption evenly.

The Low Energy Adaptive Clustering Hierarchy (LEACH) protocol^5^ is a revolutionary clustering-based routing protocol renowned for its superior energy efficiency compared to others of its time^6,7^. However, the reliance of CH on one-hop transmission for sending data to the BS has its limitations. Subsequent iterations of LEACH have implemented multi-hop transmission to improve network lifespan and energy efficiency^8^. Use of unequal clustering reduces the hot-spot problem to a certain extend^9,10^. The heterogeneous network provides superior performance and effectively in resolving the hot-spot problem^11,12^. However, it’s important to acknowledge that implementing a heterogeneous network also leads to increased network costs and complexity. In contemporary WSN, mobile elements are extensively utilized to optimize data collection efficiency and reduce energy consumption. Two prominent types of mobile elements are commonly employed: (i) mobile data collector (MDC) and (ii) mobile sink. The MDC functions as an intermediary between the sensor nodes and the BS. It collects data from individual nodes or relay nodes during its journey and transfers all gathered data to the BS upon arrival. Conversely, a mobile sink is the BS with mobility which collects data from nodes along its trajectory path and directly transmit it to the external world when necessary. These mobile elements traverse different trajectories such as random paths, fixed paths, on-demand routes and priority-based paths.

Many multi-hop clustering-based routing protocols encounter with the hotspot problem where nodes near the experience heavy data traffic resulting in shorter life span. These nodes expend much more energy in relaying data than performing their primary functions of sensing and transmitting information. As they deplete quickly, the remaining nodes are forced to transmit data over longer distances leading to accelerated energy exhaustion across the network. Additionally, the energy overhead involved in cluster formation and CH selection further strains the nodes. These challenges such as hotspot issues, long-distance transmissions and clustering energy demands collectively shorten the network’s lifespan. To address this, a new centralized protocol is proposed integrating data mules to improve network longevity. The protocol also optimizes clustering by capping node’s transmission range to a threshold based on the sensor node radio energy model, ensuring efficient energy use.

This paper introduces the Energy-Efficient Mega-Cluster-based Routing (EEMCR) Protocol, which combines two-level clustering with mobile data collector (MDC) techniques to address the hotspot problem and ensure uniform data traffic. Designed for larger sensing areas, the protocol employs a centralized, grid-based fixed clustering approach that leverages the capabilities of the BS. The BS is responsible for network division into fixed-size clusters, formation of mega-clusters (clusters of clusters), selection of CHs & MCHs, TDMA slot allocation and the definition of data mule paths. The network is divided into equal-sized, square-shaped clusters and each mega-cluster consists of four clusters. The size of each cluster is determined by the threshold transmission range of the radio energy model, ensuring nodes transmit within this range to conserve energy. Each node is assigned a unique integral number based on its expected transmission distance and nodes with the highest energy are selected as CHs in each round. One MCH is chosen among the four CHs and the role of MCH is rotated to balance the overhead across the clusters.

To further optimize data flow, two data mules alternate collecting data from the border MCHs based on the round number ensuring even distribution of data traffic. Information from CMs is communicated to the BS via intermediate CHs, MCHs and Data Mules using allocated TDMA slots.

By implementing two-level data aggregation and restricting node’s transmission range to the threshold distance, the protocol minimizes energy depletion ensuring nodes only transmit data energy efficiently at a rate of d^2^. The centralized BS performs overhead tasks like cluster formation, CH and MCH selection and rotation which significantly conserves sensor node energy. These combined strategies result in energy savings, improved network stability and an extended network lifespan.

The remaining portion of the paper is structured as follows: Section-2 provides a summary of related work, Section-3 elaborates on the system model consists of network model and energy consumption model, Section-4 outlines the proposed protocol, Section-5 presents simulation results and Section-6 concludes with a brief discussion on future work.

## Related Works

LEACH is the pioneering hierarchical routing protocol based on distributed clustering principles^5^. Operating on a round-based mechanism, each round starts with all nodes generating a random number between 0 and 1. If a node’s generated random number falls below a threshold value, it is elected as a CH of the round & subsequently broadcasting the respective message to its member nodes. Despite its energy efficiency, this protocol suffers from several drawbacks including (i) the lack of consideration for node energy in CH selection, (ii) direct single-hop transmission of data from CHs to the BS and (iii) the random placement of CHs within clusters. Consequently, various researchers have proposed solutions to address these issues^8^. Heinzelman et al.^13^ extended their previous LEACH protocol with LEACH-centralized (LEACH-C) to mitigate its drawbacks. They centralized the protocol and fixed the number of CHs for all rounds, thereby reducing the overhead associated with forming new clusters in each round. Despite showing improved performance compared to LEACH, this protocol still faces challenges such as single-hop transmission from CHs to the BS and issues with CH selection. Huamei et al.^14^ proposes an energy-efficient non-uniform clustering routing protocol in order to improve node energy efficiency and balance energy consumption in WSNs. Furthermore, a non-uniform clustering network partition is presented to optimize the cluster heads dynamical selection approach and lower the likelihood of energy hole occurrences, The simulation experiment showed that the suggested protocol and improved algorithm could increase energy efficiency by 20% and prolong network lifetime. In order to address the issue of hotspots, Zhu and Wei^15^ suggested an energy-efficient routing protocol that uses unequal clustering technology. Additionally, it suggests a double cluster head method to lower the energy consumption of the cluster heads. Furthermore, a hybrid cluster head rotation technique based on time-driven and energy-driven is proposed to balance the energy consumption between cluster heads and cluster members. This can lead to more appropriate rotation timing and efficient energy consumption. Alharbi et al. tackled two related problems, namely routing and clustering, for extensive IoT-based wireless sensor networks (WSNs)^16^. They provided a better protocol enabling both tasks to be addressed simultaneously. Area-based clustering based on the transmission range of network nodes is made possible by improved routing and clustering. Cluster heads are chosen during the clustering procedure to ensure failover-proof routing. Finding the least number of hops while having alternative routing paths available results in an efficient routing path. Reliable network topology, extended network lifetime, effective node density control, and increased total network capacity are all demonstrated by theoretical and simulation results. Hu et al. presented QPSOFL^17^, a clustering and routing protocol that combines a fuzzy logic system with quantum particle swarm optimization to improve energy efficiency and extend network lifetime. To choose the best cluster heads, QPSOFL uses an improved quantum particle swarm optimization technique that uses Sobol sequences for population diversification at initialization. The optimal next-hop cluster head within QPSOFL is chosen by a fuzzy logic system using descriptors including relay distance, energy deviation, and residual energy. Comprehensive models contrast QPSOFL’s performance with that of the similar existing protocols in terms of network lifetime, throughput, energy consumption, and scalability, showing QPSOFL to be superior to these protocols control and increased network capacity in general.

Lui et al. introduced a grid-based scheme called FCEEC^18^ to address the hot-spot issue. In this protocol, CH selection is primarily based on the transmission distance within the cluster. The most suitable CH is chosen as the relay node with their numbers being limited. This ensures balanced energy consumption with clusters nearest to the BS consuming less energy. Grid-Based Routing (DBSCAN)^19^ is another grid-based protocol that selects the node nearby the BS as CH in each cluster. However, this approach leads to increased intra-cluster communication costs and a consequent reduction in network lifetime. Jannu and Jana introduced a novel grid-based routing algorithm named Low Power Grid-based Cluster Routing Algorithm (LPGCR)^20^, primarily aimed at eliminating the hot-spot problem. They partitioned entire network into equal grids and selected the node with the maximum remaining energy as the CH in each grid. However, the limitation of single-hop communication between the CH and the BS restricts its applicability in large-area. Gupta et al. introduced an upgraded version of distributed unequal clustering based on energy awareness named EADUC^10^. This scheme is designed to address hot-spot problems using multi-hop transmission. CHs are selected based on the BS location, remaining energy and node degree. Relay nodes for multi-hop communication are chosen based on their distance from the CH and energy expenditure. However, the scheme’s heterogeneity increases overall cost and complexity posing a primary challenge. 

Shah et al.^21^ proposed a new reference communication model consists of three layers. The lower layer comprises of numerous sparsely deployed sensor nodes. The in-between layer involves mobile data collector known as MULES which traverse the network randomly to gather data from the sensors. Subsequently, the collected data from these MULES is then transmitted to the upper layer access point, which is IP-enabled and interconnected with the wider network. This model operates similar to an opportunistic network characterized by increased delays and uncontrolled behavior of MULEs. Singh and Kumar^22^ presented a comprehensive survey to examines how mobile element trajectory optimization contributes to WSN performance enhancements. In the survey, three key criteria have been employed; applications, trajectory approaches, and domains utilized in the trajectory formulation. Numerous plans and their sub-aspects are covered under these three headings. Eight key parameters—such as the trajectory pattern, quantity of mobile elements, speed, type of mobile element, etc. are compared and shown chronologically. Singh et al.^23^ proposed a grid-based cluster routing scheme featuring mobile mules. They utilized an odd–even level routing strategy for data forwarding within each level, while the mobile mules gathered data from the boundary Cluster Heads (CHs) of each level. Despite utilizing two mules, only one mule visits a level at a time based on round numbers. However, it may not be suitable for large-area networks due to its centralized clustering approach. Furthermore, they have extended on this work by using a load balancing virtual level-based routing (LBVLR)^24^ approach that deals with energy holes and ensures efficient data transport by utilizing a data mule as a mobile carrier. This scheme applied horizontal level routing in both left and right directions in an effort to alleviate the energy hole issue. With the aid of a mobile data mule and the BS, fixed virtual rectangular grids of uniform size are created that reduce network overheads and result in significant energy savings. This method employs three distinct data gathering strategies, which we refer to as the uniform speed of the mule, variable speed of the mule and sojourn point of the mule based on the mule’s speed during the trajectory. Kaswan et al.^25^ proposed two new techniques, EDT and DAEDT, for data collection utilizing the controlled mobility of mobile sinks. Rendezvous points are determined according to the energy density of each sensor node in a flat network where one-hop communication is prioritized. In EDT, the algorithm calculates the impact of every other node within communication range on a sensor node, selecting lowest impact nodes as Rendezvous Points (RPs). A subset of RPs that covers all deployed nodes is identified and a Traveling Salesman Problem (TSP) path covering these RPs is established. However, EDT doesn’t think of the delay bound linked to the application, rendering it unsuitable for applications requiring timely responsiveness. The worst-case time complexity of this algorithm is O (${n}^{3}$), where n represents the number of deployed sensors. The TSP path in DAEDT is constrained by a user-defined delay limit, resulting in fewer RPs and increased communication distance. Consequently, when considering delay, sensors can no longer strictly communicate within a 1-hop distance, leading to higher energy consumption. Singh et al.^23^ expanded their earlier scheme by incorporating unequal clustering with mobile mules having various trajectory paths^26^. They enhanced the scheme and devised a method to compute an optimal Cluster Head (CH) change factor and a reduction factor for the cluster’s size. Mehra et al.^27^ introduced Fuzzy-based Enhanced Cluster Head Selection (FBECS) algorithm which utilizes fuzzy logic to select cluster heads in WSNs. FBECS employs fuzzy logic to evaluate candidate nodes for cluster head selection based on multiple criteria such as remaining energy, distance to BS & communication quality. By considering these factors holistically and incorporating expert knowledge through fuzzy rules, FBECS aims to select optimal cluster heads that can effectively manage network resources and prolong network lifetime. Sahoo et al.^28^ proposed an intelligent clustering algorithm designed to mitigate the impact of uncertainty on WSN lifetime. The algorithm employs intelligent techniques, such as fuzzy logic or machine learning, to analyze environmental data and node characteristics allowing nodes to make informed decisions regarding cluster formation and operation. By considering uncertainty factors, such as node reliability and environmental variability, the algorithm aims to optimize energy usage and prolong network lifetime The paper^29^ proposes an energy-efficient algorithm called the Electrostatic Discharge Algorithm (ESDA) for selecting cluster heads and establishing short routing paths within the network. It employs a method for selecting cluster heads based on their residual energy levels, communication distance and network density. It establishes short routing paths between sensor nodes and cluster heads to reduce transmission energy and latency.

The proposed protocol is compared with existing protocols such as FBECS, DBSCAN, LPGPR and FCEEC based on several key parameters. The FBECS protocol has limitations like computational overhead from fuzzy logic, scalability issues in large or dynamic networks. Also, increased energy and communication costs due to frequent CH evaluations and data collection which can negatively impact efficiency and network lifespan. The DBSCAN protocol faces high intra-cluster communication costs, uneven energy depletion and reduced network lifetime due to static CH selection near the BS. It also struggles with scalability, lacks adaptability to dynamic environments and does not balance cluster loads effectively. LPGPR has issues with energy imbalance, scalability and high communication overhead, especially in large or dense networks. It struggles with fixed cluster formations, limited load balancing and inadequate handling of node failures. The FCEEC protocol has limited adaptability in dynamic networks, energy depletion in relay nodes, potential cluster size imbalances, scalability issues, communication overhead and inflexibility due to its reliance on transmission distance for CH selection. The proposed protocol addresses and overcomes these limitations aiming to improve energy efficiency, scalability, adaptability and network reliability.

## System Models

### Network Model

All sensors are randomly distributed throughout a vast area in our network model. SN = {SN_1_, SN_2_, SN_3_,…,SN_N_} represents the collection of *N* sensor nodes deployed within the network, where the ${i}_{th}$ sensor is identified as SN_i_.

There are few assumptions considered in designing our protocol. our network assumptions include homogeneity among nodes with unique IDs and initial energy levels. The Base Station (BS) remains stationary outside the observation area, while sender sensor nodes adjust their transmission power based on distance from the destination node. The BS boasts infinite computation and communication abilities and possesses information on node ID and location. Data Mules are mobile devices without power constraints and all nodes can switch between active and sleep modes as needed.

### Energy Consumption Model

The framework outlined in^14^ is adopted by our network’s radio energy model where the energy usage of transmitting node is dependent on its proximity to the receiving node.

The energy needed to communicate “p” bits of information across a range x is expressed as:

1 $${E}_{T}\left(p,x\right)=\left\{ \begin{array}{c}\left( {E}_{el}*p+p*{\in }_{fs}*{x}^{2}\right), x<{d}_{0}\\ \left( {E}_{el}*p+p*{\in }_{mp}*{x}^{4}\right), x\ge {d}_{0} \end{array}\right.$$

The threshold distance *d*_0_ is calculated as

2 $${ d}_{0}=\sqrt{\frac{{\in }_{fs}}{{\in }_{mp}}}$$

The energy expended by the sensors in receiving *p* bits of information is represented as

3 $${ E}_{R}\left(p\right)=\left( {E}_{el}*p\right)$$

The energy expended by the sensors in aggregating *m* redundant information, each composed of *p* bits, is described as:

4 $${ E}_{DA}\left(m,p\right)= \left({E}_{agg}*p*\text{m}\right)$$

where ${E}_{el}\,\&\,{ E}_{agg}$ signifies electronic energy and aggregation energy of a sensor per bit of data transmitted and aggregated respectively.

${\in }_{fs}\,\&\,{\in }_{mp}$ signifies amplifier energy of free space & multi-path fading model respectively.

The radio energy model of a sensor node estimates the energy consumption of sensors during communication tasks like data transmission, reception, aggregation etc. The estimated energy depletion of sensors in performing various tasks can be calculated by using Eqs. 1, 3 and 4.

Equation 1 calculates the expected energy loss of sensor in transmitting data over a distance. The calculated energy is directly proportional to the distance over which data needs to transmitted. Energy consumption increases with the square of the distance (d^2^) for shorter distances and with the fourth power of the distance (d^4^) for longer distances. This indicates that sensors consume energy much faster over longer distances compared to shorter ones. The distance up to which transmission is energy efficient, is known as threshold distance or range of radio energy model. The value of threshold range ${d}_{0}$ is calculated by using the Eq. 2.

By restricting sensor nodes to transmit only within the threshold range, the overall energy depletion is minimized, prolonging the lifespan of individual sensors and extending the life of the entire network.

## Proposed Protocol

The proposed protocol utilizes a fixed clustering approach where the CH is selected based on the maximum residual energy and distance from cluster nodes centroid in each round. It operates in a fully centralized manner with the BS handling cluster formation, CH selection and TDMA schedule creation for every round. To balance energy consumption and manage data traffic, two data mules collect information from the boundary-area MCHs within the network.

The protocol is divided into three main phases: network division, setup and steady operation. The network division phase occurs once during the initial deployment of sensors in the network area. Subsequent operation is structured into rounds, each consisting of a setup phase followed by a steady phase.

Initially, the network is divided into a fixed number of square-shaped clusters based on the sensor radio energy model’s threshold transmission distance *d*_0_, ensuring that each node’s transmission distance remains within *d*_0_. Once clusters are formed, each cluster member is assigned a unique integer, starting from 0 in ascending order. This assigned number remains fixed throughout the network’s lifetime and is determined based on the node’s expected transmission range in future rounds. Using this numbering scheme, a cluster member is elected as the Cluster Head (CH) in each round ensuring that the CH is always the node with the highest residual energy.

This CH selection method addresses the shortcomings of the LEACH protocol where low-energy nodes might become CHs after a few rounds. Four adjacent clusters are grouped to form a mega-cluster and one CH among the four is designated as the MCH for that round. The roles of CH and MCH are evenly distributed among the nodes across the network to balance workload.

The protocol implements two levels of data aggregation: the CH aggregates data within its cluster and the MCH further aggregates data for its mega-cluster. This dual aggregation reduces the network’s data flow rate and extends its lifetime. Data mules collect aggregated data from either the leftmost or rightmost MCHs based on the odd or even round number. This approach ensures uniform energy usage and balanced data traffic across the network limiting transmission distances to the threshold value and mitigating the hotspot problem. By integrating fixed clustering and the proposed CH selection scheme, the protocol achieves improved network lifetime compared to existing routing protocols.

### Network Division Phase

The network division phase begins after sensor deployment and involves partitioning the entire region into a fixed number of mega-clusters (grids) of equal dimensions which remain constant throughout the network’s lifetime. Initially, the area is divided into* n* horizontal rectangular lanes of equal width *w*, referred to as “levels”. These levels are sequentially labelled with IDs ranging from* 0* to *n-1*, starting from the top and proceeding to the bottom.

Next, vertical lines perpendicular to these levels divide the horizontal lanes into square grids known as mega-clusters, each with an edge length of *w*. Each mega-cluster is further subdivided into four smaller square-shaped clusters with an edge length of $w/2.$ Unique identifiers ${M}_{ij}$​ are assigned to the mega-clusters, where* i* represents the level number and* j* denotes the vertical rectangular lane number. Similarly, unique identifiers ${C}_{ijk}$ are assigned to individual clusters where $ij$ corresponds to the mega-cluster ID and $k$ indicates the cluster number within that mega-cluster. This structured partitioning ensures efficient organization and management of the network for subsequent operations.

**Fig. 1:**
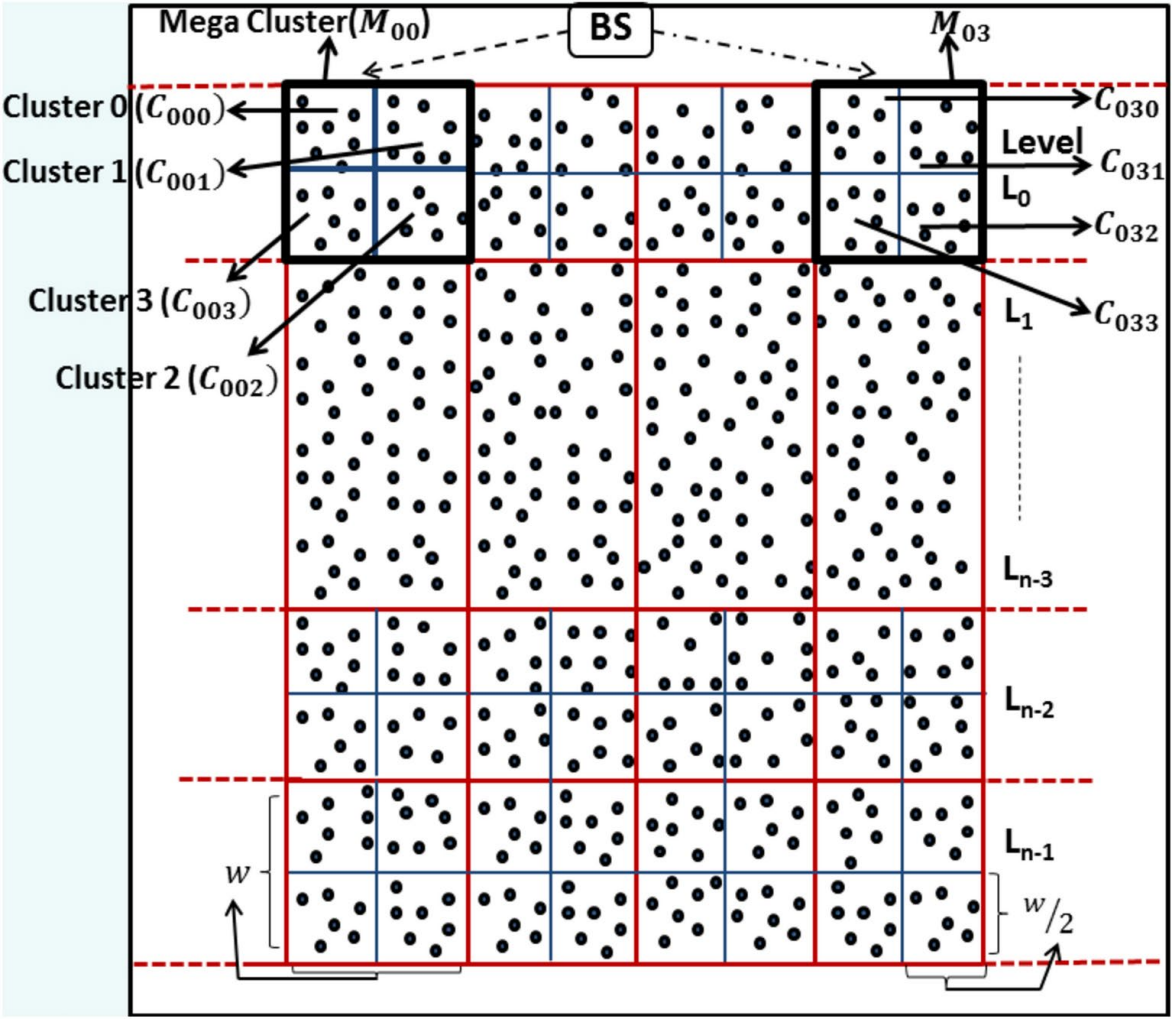
Network division phase of proposed protocol.

Figure 1 illustrates the segmentation of clusters and mega-clusters within the network. Algorithm-1 provides an overview of the processes involved in this phase. The calculation of the mega-cluster width $w$ ensures that the potential transmission range for any interaction (i.e., MCH to MCH, CH to MCH and CM to CH) in the sensing region consistently remains below the sensor energy consumption model threshold distance ${d}_{0}$. Three types of communication are possible in the network—CM to CH, CH to MCH and MCH to MCH. From Algorithm-4, we can see that the protocol is designed in such a way that the CH of same cluster number act as MCH for all the mega-clusters for a round. For example, if CH of cluster number 3 of a mega-cluster act as MCH for a round then CH of cluster number 3 of all the mega-cluster act as MCH for that round.

**Fig. 2:**
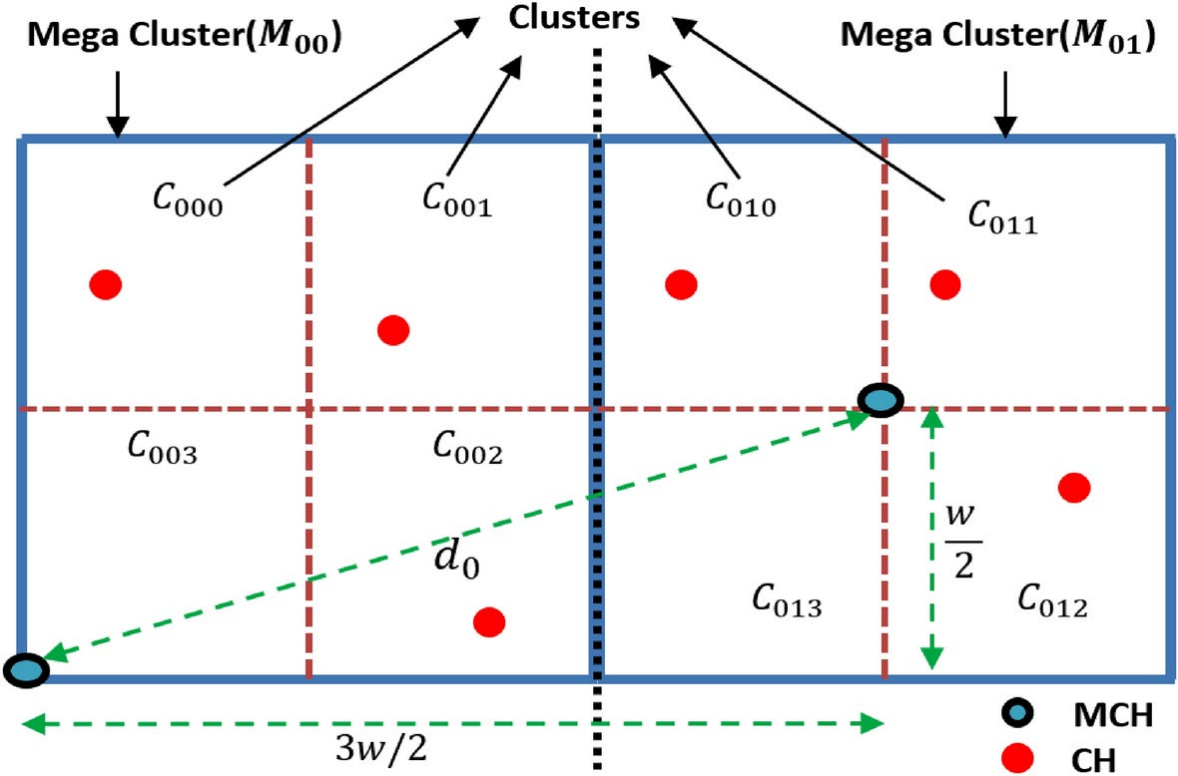
Communication between two adjacent MCHs in a level.

We are considering a network structure with MCHs and CHs communicating within their respective clusters. The scenario uses specific geometric relationships to ensure that communication remains energy efficient and the transmission distance does not exceed a threshold value that would lead to excessive energy usage. Figure 2 shows communication between two adjacent MCHs within a level under the worst-case scenario, wherein the two MCHs are positioned at diagonally opposite corners of the rectangular area. Considering this diagonal distance as threshold transmission distance ${d}_{0}$, BS calculates the width of the mega-cluster for network division. Equation 5 shows the width of the mega-cluster *w* in terms of the threshold transmission distance ${d}_{0}.$ This equation calculates the diagonal distance between two MCHs located at diagonally opposite corners of the rectangular area of the mega-cluster.

$${\text{Here}},\quad d_{0} = \sqrt {\left( \frac{3w}{2} \right)^{2} + \left( \frac{w}{2} \right)^{2} }$$

5 $${\text{Therefore}},\quad w = \frac{{2d_{0} }}{{\sqrt {10} }}$$

**Fig. 3:**
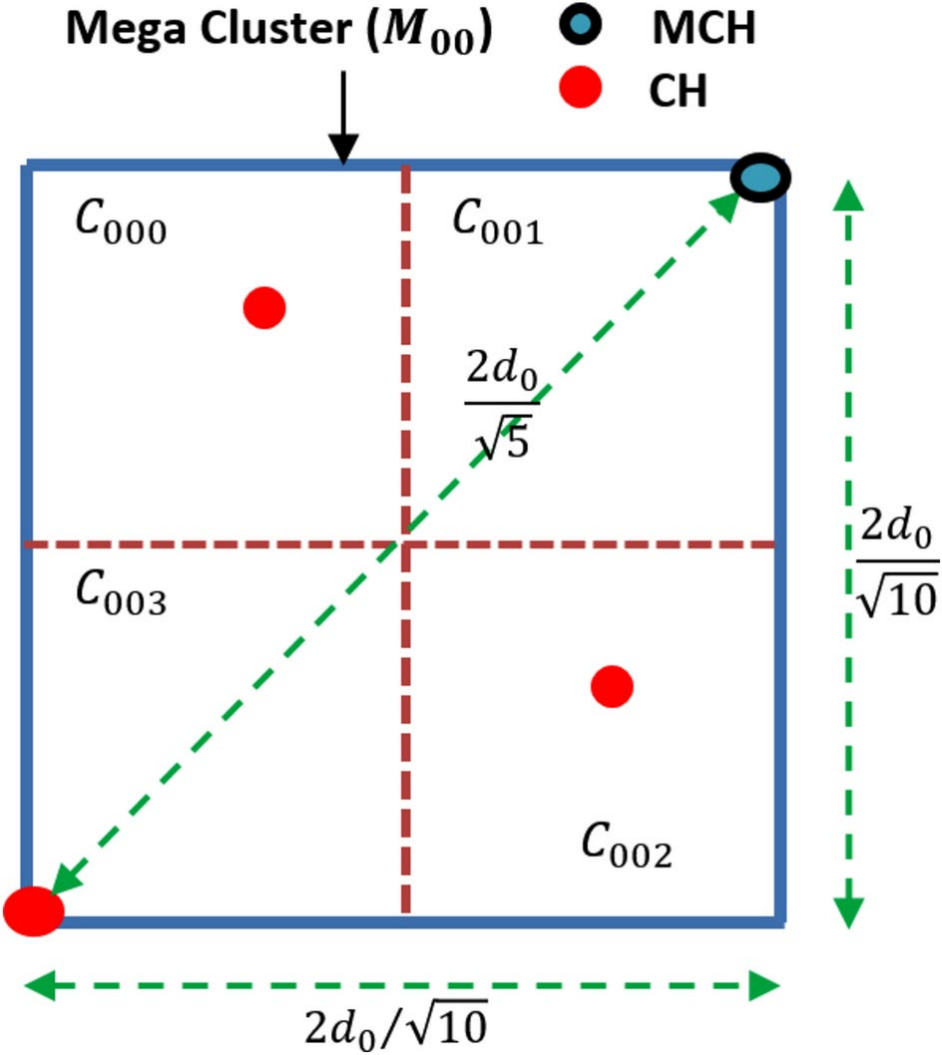
Communication within a mega-cluster.

Figure 3 describes the communication scenario between the MCH and CH within a mega-cluster. In the worst-case scenario, both the MCH and CH are positioned at diagonally opposite corners of the mega-cluster. Given that the size of the mega-cluster is $2{d}_{0}/\sqrt{10}$, we now consider the maximum distance between the MCH and CH within the mega-cluster. The maximum distance for communication between the MCH and the CH within the mega-cluster is given by $2{d}_{0}/\sqrt{5}$. This distance is less than the threshold transmission distance ${d}_{0}$, which ensures that the communication remains within the range that is optimal for energy efficiency and network performance.

Hence, the network division strategy is validated by the derived equations to ensure the energy reduction of nodes at a rate of ${d}^{2}$. The width of the mega-cluster $2{d}_{0}/\sqrt{10}$ ensures that communications within the cluster will always stay within the threshold distance ${d}_{0}$ preventing excessive energy consumption. Additionally, the maximum communication distance between the MCH and CH ($2{d}_{0}/\sqrt{5}$ ) is less than ${d}_{0}$, confirming that the network is designed for efficient data transmission and energy usage.

**Algorithm 1:**
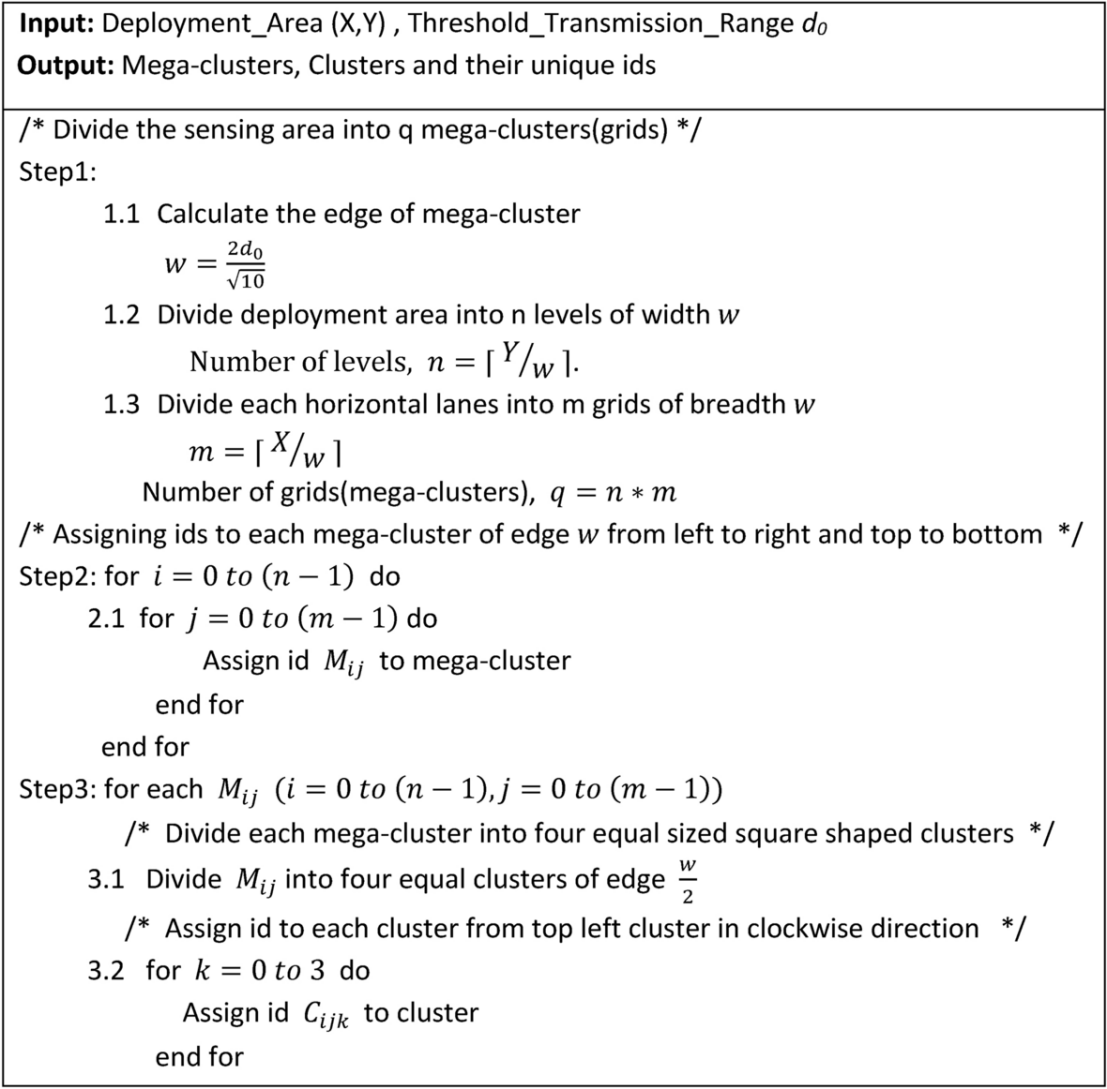
Network Division of Sensing Area.

### Setup Phase

The setup phase of the EEMCR protocol comprises three key algorithms executed at the BS:

- **Algorithm-2** assigns numbers to all nodes in the network.
- **Algorithm-3** selects Cluster Heads (CHs) for each round.
- **Algorithm-4** determines the Master Cluster Head (MCH) for each mega-cluster among its four CHs.

#### Algorithm-2: Node Number Assignment

Algorithm-2 is a prediction algorithm that establishes a pattern for CH selection in subsequent rounds. It estimates the energy depletion of each node based on its expected transmission distance for each round. This algorithm is executed only once during the network’s lifetime.

1. The BS begins by selecting a cluster and determining the total number of nodes within it.
2. It calculates the centroid of the selected cluster based on the node positions.
3. The BS computes the distance of each node from the cluster’s centroid.
4. Nodes are assigned numbers sequentially starting with N = 0 for the node nearest to the centroid.
5. The algorithm then computes the distances of the remaining nodes from the node with the highest assigned number. The nearest unassigned node is selected and assigned the next number N + 1.
6. This process continues until all nodes within the cluster have been assigned a number.

The algorithm is repeated for every cluster in the network and the BS maintains a comprehensive table of the assigned numbers. This table is critical for Algorithm-3 which uses it to select CHs in each round.

**Algorithm 2:**
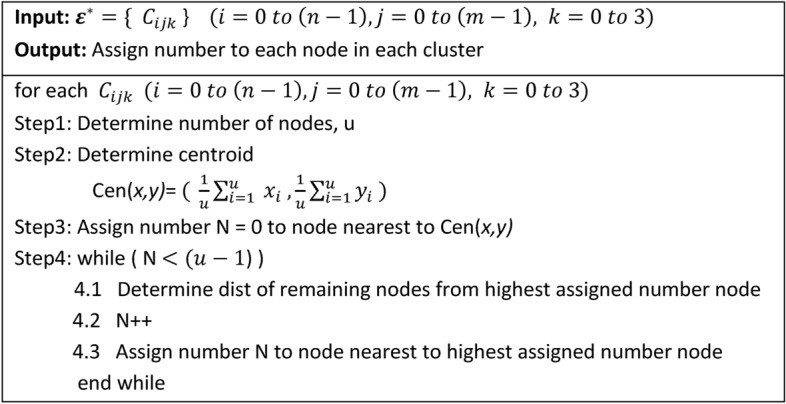
Number Assignment to Nodes.

#### Role of Algorithm-2 in CH selection

Algorithm-2 predicts the relative energy loss of nodes within each cluster based on their expected transmission distances, using a mathematical model (explained in Eq. 1). This ensures that CH selection prioritizes nodes with the maximum residual energy for each round. For instance:

- In Round 0, all nodes with N = 0 are selected as CHs for their respective clusters.
- After Round 0, the node closest to the current CH (N = 1) is likely to have the highest residual energy due to minimal energy depletion. This node becomes the CH for Round 1.
- The process continues iteratively in subsequent rounds ensuring energy-efficient CH rotation.

#### Output of Algorithm-2

The output of Algorithm-2 is a structured pattern of node numbering that ensures nodes with the least energy depletion are selected as CHs in future rounds. This approach balances energy consumption among all nodes within a cluster prolonging network lifetime.

#### Visualization

**Fig. 4:**
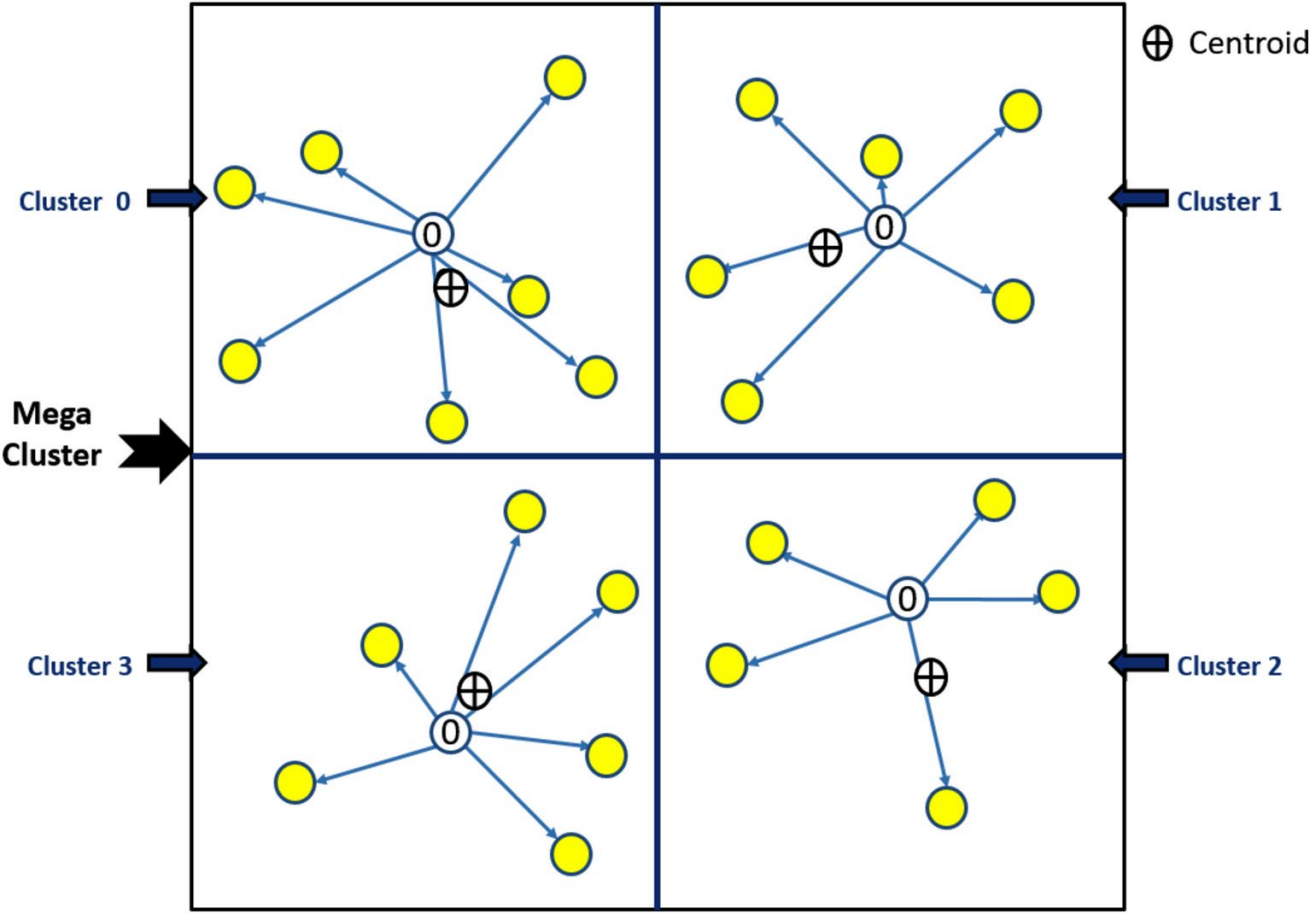
Shows working of Algorithm 2 and assignment of number 0 to the node nearest to centroid of each cluster.

**Fig. 5:**
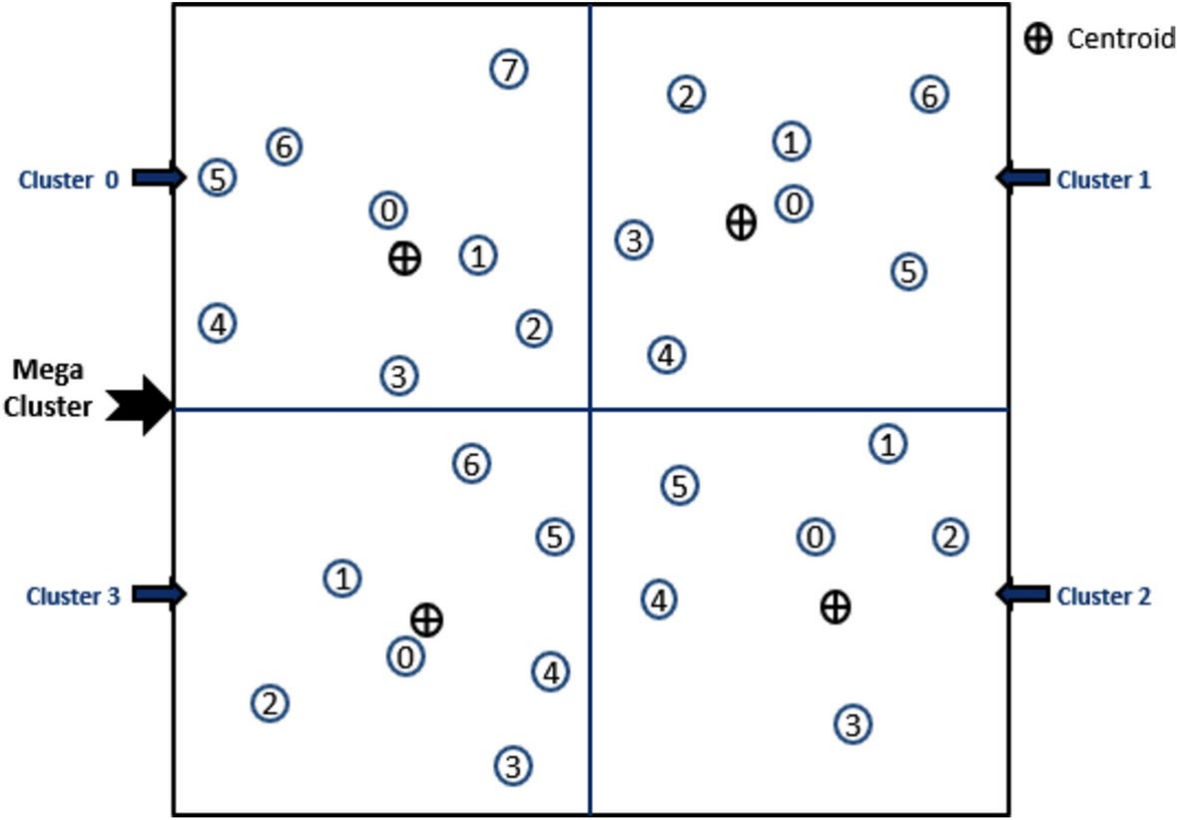
Shows the number assigned to each node in the cluster after the complete execution of Algorithm-2.

Figures 4 and 5 illustrate the workflow and results of Algorithm-2 showcasing the sequential numbering process and how it informs CH selection. This systematic approach ensures that the node with the maximum residual energy is consistently chosen, enhancing the network’s energy efficiency.

#### Algorithm-3: Cluster Head (CH) Selection

Algorithm-3 is executed by the BS at the start of each round to determine the CH for every cluster. The algorithm leverages the numbers assigned to nodes during Algorithm-2 and operates based on the cluster size and the energy status of the nodes.

#### Process overview

1. **Node Record Maintenance:**
2. Before Round 0, the BS maintains a comprehensive record for each node which includes: Energy level, Position coordinates, Cluster ID and mega-cluster ID, Number assigned to the node (determined by Algorithm-2)
3. This record is updated at the end of every round by calculating the energy depleted during: Data reception, Data aggregation, Data transmission

The calculations are based on predefined equations (Eqs. 1, 3, 4).

2. **CH Selection Logic:**
3. At the beginning of each round, the BS selects the node with the maximum residual energy within each cluster as the CH for that round. This ensures energy-efficient CH rotation and prevents premature node depletion.
4. The frequency of CH role rotation depends on: assigned numbers which establish a sequence and number of nodes within the cluster.
5. **Centralized Execution:**
6. Since the BS maintains up-to-date information for all nodes, the CH selection process eliminates the need for nodes to broadcast overhead messages such as: Energy status updates, Alive status signals and Cluster formation packets.

This centralized approach reduces energy depletion associated with these overhead tasks.

#### Advantages

- **Energy Conservation:** The centralized management of node records eliminates redundant communication overhead, saving significant energy for all nodes.
- **Efficiency in CH Selection:** The BS uses a well-maintained record to consistently select the node with the highest residual energy, ensuring that CH selection is optimal in each round.
- **Reduced Complexity:** Nodes are freed from performing energy-intensive tasks such as broadcasting and coordinating CH selection, allowing them to conserve energy for critical operations.

By combining centralized record-keeping and systematic CH selection, Algorithm-3 ensures efficient energy usage and prolongs the network’s operational lifetime. This methodology also supports seamless coordination among clusters, paving the way for scalable and reliable network operation.

#### Algorithm-4: Master Cluster Head (MCH) Selection

Algorithm-4 is executed at the start of each round to select the MCH for each mega-cluster. Since the MCH is also the CH for its own cluster, it processes a larger volume of data compared to other nodes. The algorithm is designed to distribute the additional workload of the MCH role uniformly among all nodes over time ensuring fair energy usage across the network.

#### Functionality

1. **Role Assignment:** At the beginning of each round, Algorithm-4 determines which cluster’s CH within each mega-cluster will act as the MCH for that round.
2. **Cluster Rotation:** The algorithm rotates the MCH role among the four clusters within a mega-cluster in a cyclic manner.

For example:

- In Round 0, the CH of cluster 0 in each mega-cluster serves as the MCH.
- In Round 1, the CH of cluster 1 becomes the MCH.

This rotation continues sequentially ensuring that no single cluster is disproportionately burdened with the MCH role.

3. **Uniform Workload Distribution:** By systematically rotating the MCH responsibility, the algorithm ensures that the additional processing and communication workload of the MCH is shared equally among all nodes over time.

**Algorithm 3:**
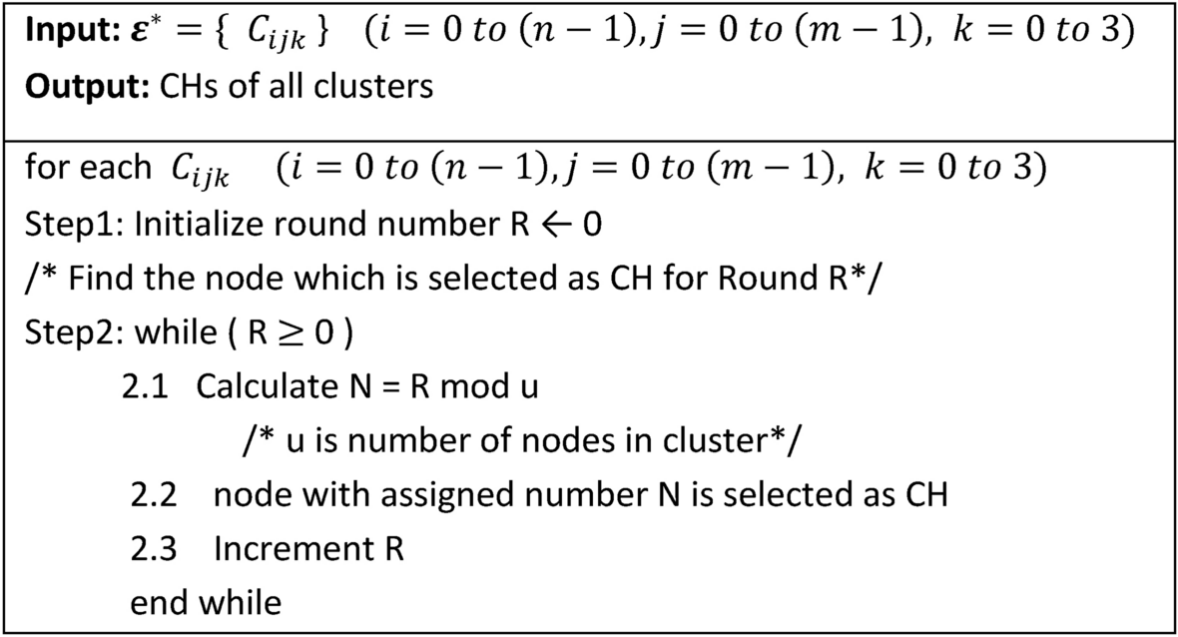
Cluster Head Selection.

**Algorithm 4:**
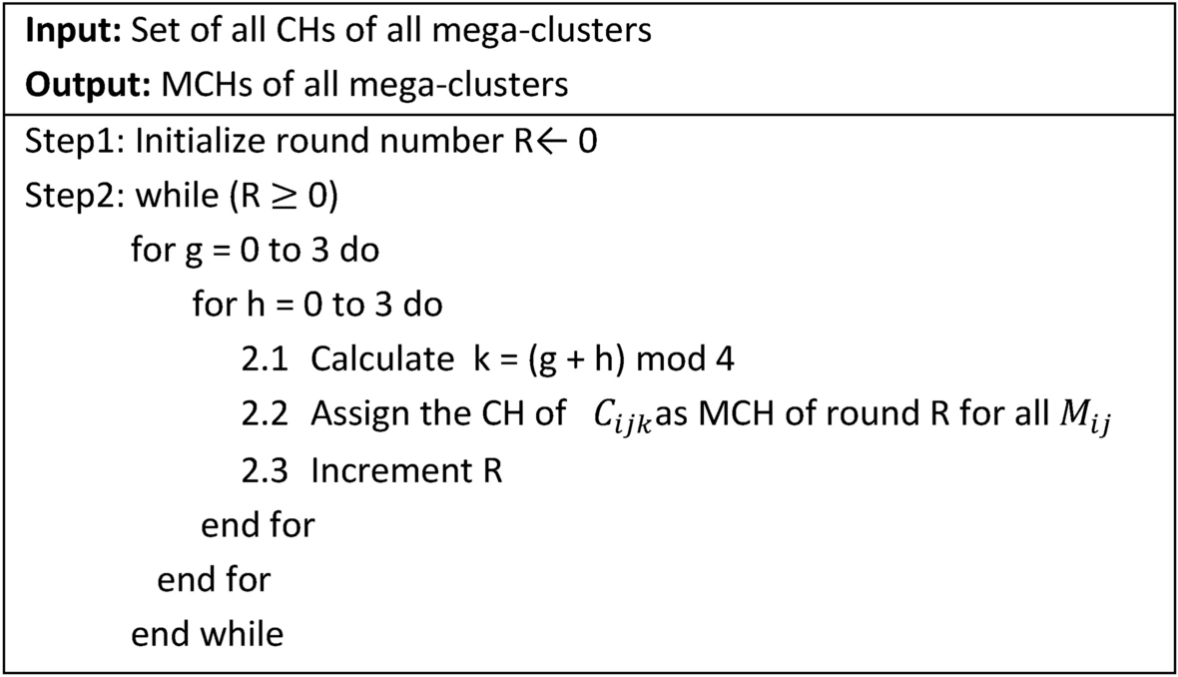
Mega-Cluster Head Selection.

#### Illustration of MCH role rotation

**Fig. 6:**
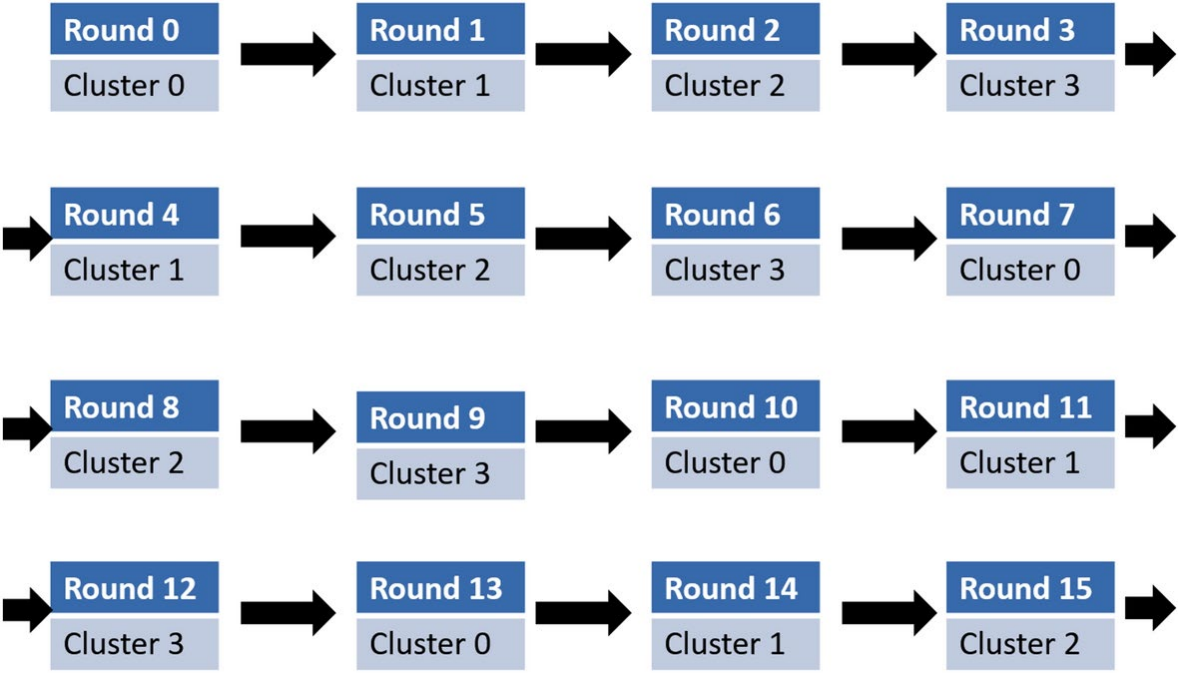
shows the rotation of role of MCH among CHs within mega-cluster.

Figure 6 visually demonstrates how the MCH role shifts from one cluster to another in each round. For example, if the algorithm specifies **cluster 3** for a particular round, the CH of cluster 3 within each mega-cluster also takes on the MCH role for that round.

#### Benefits

- **Energy Efficiency:** By rotating the MCH role, the algorithm prevents energy depletion in any single cluster or node, thereby enhancing the network’s overall lifespan.
- **Balanced Load Distribution:** Uniform workload sharing ensures that all clusters contribute equally to the additional responsibilities of being an MCH.
- **Scalability:** The cyclic selection mechanism can be easily scaled to larger networks with more mega-clusters or nodes.

Algorithm-4’s systematic approach to MCH selection plays a critical role in maintaining the network’s efficiency and prolonging its operational lifetime.

### Steady Phase

#### Data Transmission and Aggregation

During the steady phase, the nodes transmit sensed data to the BS through a multi-level hierarchical routing system. The process involves several steps to ensure efficient data transmission, aggregation and routing across the network.

1. **TDMA Slot Allocation**

- The BS identifies the cluster in the network that contains the maximum number of nodes and determines this number (u) for the cluster.
- Based on u, the BS allocates equal TDMA time slots to every CM across all clusters in the network. This ensures fair access to communication slots for all nodes.
- Each CM transmits its sensed data to its respective CH within its assigned time slot.

2. **Data Aggregation at CH**

- Once all CMs have transmitted their data, the CH aggregates the received data to reduce redundancy and minimize data size.
- The aggregated data is then forwarded to the MCH of the corresponding mega-cluster during the MCH’s allocated time slot.

3. **Data Aggregation at MCH**

- The MCH receives data from all the CHs within its mega-cluster and further aggregates it.
- The MCH then forwards the aggregated data to its neighbouring MCH within the same horizontal level (mega-cluster row).

4. **Routing of Aggregated Data**

- The direction of data flow depends on the round number:
- For odd-numbered rounds, data is routed towards the MCHs at the leftmost boundary of the network.
- For even-numbered rounds, data is routed towards the MCHs at the rightmost boundary.
- This alternating routing strategy helps balance energy consumption across the network and prevents hotspots.

5. **Communication with Data Mules**

- Data mules are pre-scheduled to visit specific access points (APs) near the boundary-area MCHs (either leftmost or rightmost, depending on the round).
- The MCHs at the boundary deliver the aggregated data to the data mules, which then transport it to the BS.

#### Key advantages

- **Energy Efficiency:** The hierarchical aggregation process reduces the total volume of data transmitted, conserving energy at every level of the network.
- **Balanced Energy Usage:** Alternating data routing directions in odd and even rounds ensure uniform energy consumption across MCHs and mega-clusters.
- **Scalable Design:** The use of data mules and hierarchical aggregation supports efficient operation even in large-scale networks.

This steady-phase methodology ensures that data flows reliably to the BS while minimizing energy consumption and preventing node overloading, thereby extending the overall network lifetime.

Total time required for sending data from CM to BS for a round is determined by

$${T}_{Round}=({u-1)T}_{CH-CM}+3*{T}_{MCH-CH}+\left(m-1\right){*T}_{MCH-MCH}+\frac{Y}{v}+n*{T}_{mule-MCH}$$

where ${T}_{CH-CM}$ is the duration within which cluster member can transmit its data to the CH.

${T}_{MCH-CH}$ is the duration within which CH can transmit its data to the MCH.

${T}_{MCH-MCH}$ is the duration within which MCH can transmit its data to the neighbor MCH.

${T}_{mule-MCH}$ is the duration within which data mule receive data from MCH at AP.

$m$ is the number of mega-clusters in a level.

$v$ is the speed of data mule.

#### Algorithm-5: Data Mule Operation and Odd–Even Round Scheme

The proposed protocol employs two data mules that alternate in receiving data from Master Cluster Heads (MCHs) based on the round number. The movement and operation of the mules are designed to balance energy consumption across the network and ensure efficient data transmission.

1. **Data Mule Operation**

- **Predefined Path:** Each data mule moves along a fixed vertical path located just to the left or right of the network area.
- **Anchor Points (APs):** The mule stops at predefined anchor points to collect aggregated data from the MCHs of boundary-area mega-clusters.

**Constant Speed and Sufficient Capacity:**

- The mules travel at a constant speed and have enough memory and energy resources to collect all aggregated data from the MCHs.
- Once the data is collected, the mule delivers it to the Base Station (BS) within a designated time interval.

**Algorithm 5:**
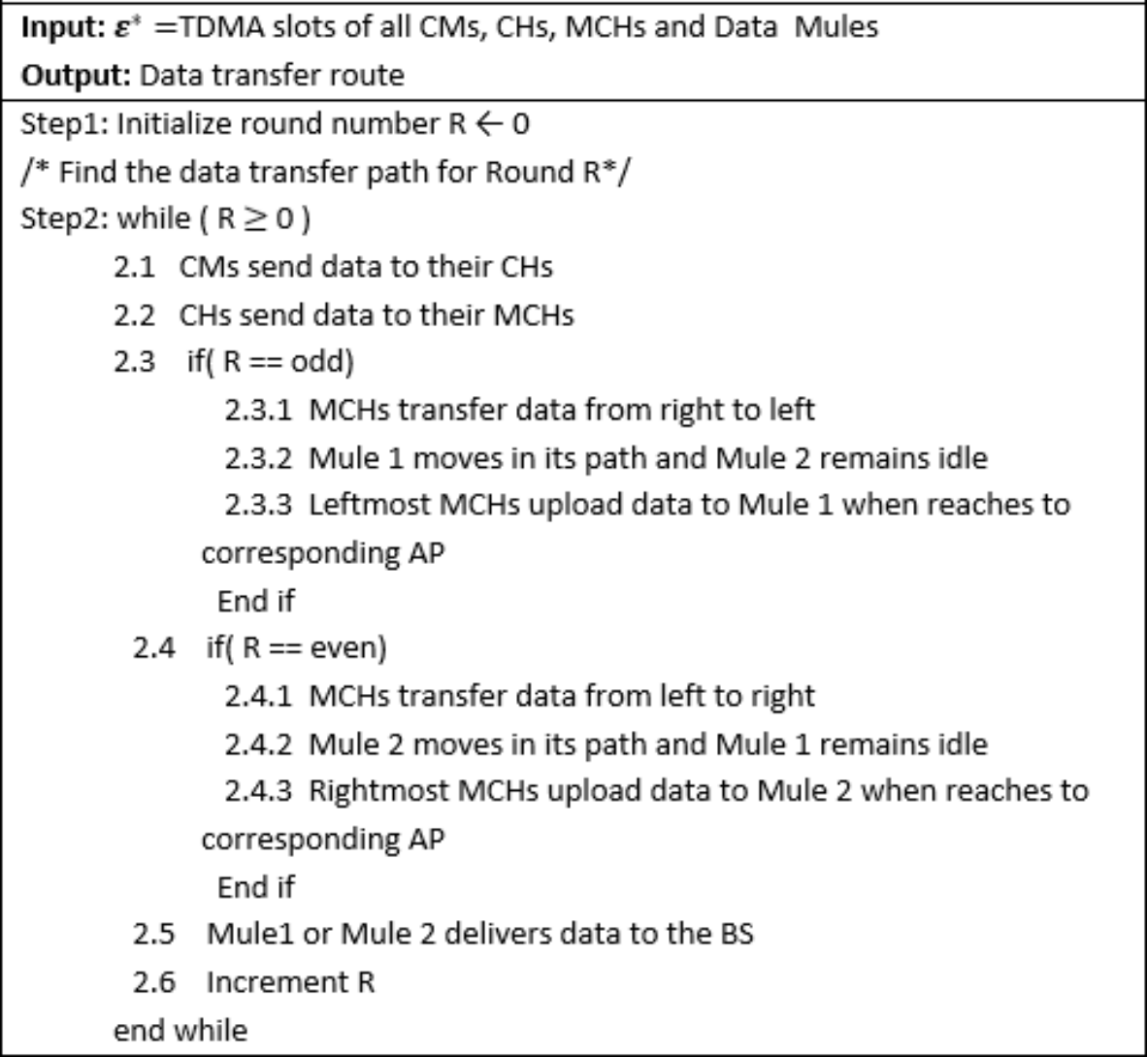
Level Routing

2. **Odd–Even Round Data Transmission Scheme**

**Odd Rounds:**

- Data is transmitted from the rightmost MCHs to the leftmost MCHs within the same level.
- The aggregated data flows through intermediate MCHs sequentially until it reaches the leftmost MCHs, where Mule 1 collects the data at its APs and delivers it to the BS.

**Even Rounds:**

- Data flows in the opposite direction, from the leftmost MCHs to the rightmost MCHs within the same level.
- The data is collected by Mule 2 at APs near the rightmost boundary and delivered to the BS.

3. **Data Aggregation and Uniform Energy Consumption**

**Data Handling in Mega-Clusters:**

- For example, if a level contains six mega-clusters:
  - In odd rounds, the rightmost mega-cluster handles its own cluster data plus the aggregated data of the other five mega-clusters.
  - In even rounds, the leftmost mega-cluster assumes this responsibility, while the rightmost handles only its own data.
- This alternating scheme ensures that both the leftmost and rightmost mega-clusters process approximately the same total amount of data after two rounds.

**Energy Depletion Uniformity:**

- By alternating the routing direction and distributing the workload, the odd–even scheme ensures that energy consumption across nodes is evenly balanced over time.
- This approach mitigates the hotspot problem, where nodes near frequently used data paths might deplete their energy faster.

4. **Benefits of the Proposed Scheme**

- **Scalability:** The use of mules allows efficient data collection even in large-scale networks.
- **Hotspot Mitigation:** Alternating the data routing direction prevents overloading specific mega-clusters or nodes, ensuring uniform energy depletion.
- **Network Longevity:** By balancing the workload among all mega-clusters and nodes, the protocol extends the overall operational lifetime of the network.
- **Efficiency:** The predefined paths and constant-speed movement of the mules minimize delays and ensure reliable data delivery.

#### Illustration

**Fig. 7:**
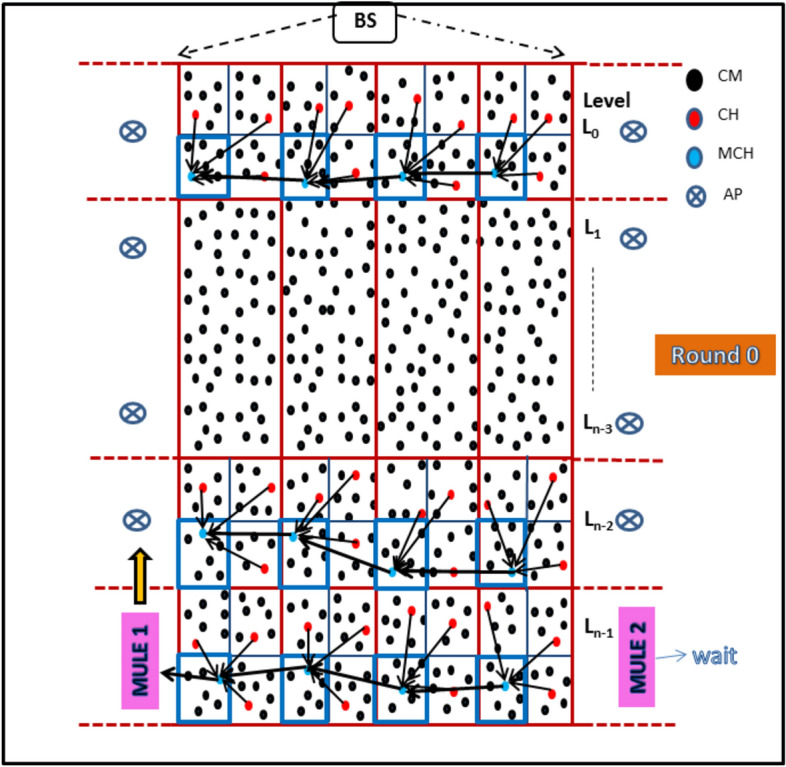
Route of data transmission in the network for an odd round in subsequent rounds in steady phase.

As depicted in Figure 7, the data mule operation and odd–even scheme provide a clear visualization of the data transmission process from cluster members to the BS. This includes the hierarchical routing of data from nodes to CHs, CHs to MCHs, and MCHs to the mules, illustrating the uniform energy depletion and balanced load distribution achieved by the protocol.

## Implementation and Results

This section provides an evaluation of the proposed work in comparison to existing protocols. The first sub-section presents an outline of the simulation environment including the simulation tool and the parameters employed in the simulations. The second sub-section explains results obtained and its comparison with existing ones under different parameters.

### Simulation Environment

The proposed protocol underwent testing using the simulation tool OMNeT + + ^30^ on a system equipped with an Intel Core i7 processor, 3.6 GHz CPU, and 16 GB RAM, operating on the Microsoft Windows 10 Professional platform. For the simulations, a deployment area of 300 × 300 square meters was considered, with 600–1200 nodes randomly distributed. The parameter values utilized are listed in Table 1.

**Table 1:** Simulation Parameters.

Network _Area (in m^2^)	300 * 300
Threshold_Dist ${\text{d}}_{0}$(in m)	87
Num_of_clusters	36
Num_of_Nodes	600 & 1200
Initial_Energy_of_Node (in J)	1.0
${\text{E}}_{\text{el}}$(in nJ/bit)	50
${\text{E}}_{\text{agg}}$(in nJ/bit)	5
${\text{e}}_{\text{fs}}$(in pJ/bit/m^2^)	10
${\text{e}}_{\text{mp}}$(in pJ/bit/m^2^)	0.0013
Data_Packet_Length (in bits)	1000
Control_Packet_Length (in bits)	

**Fig. 8:**
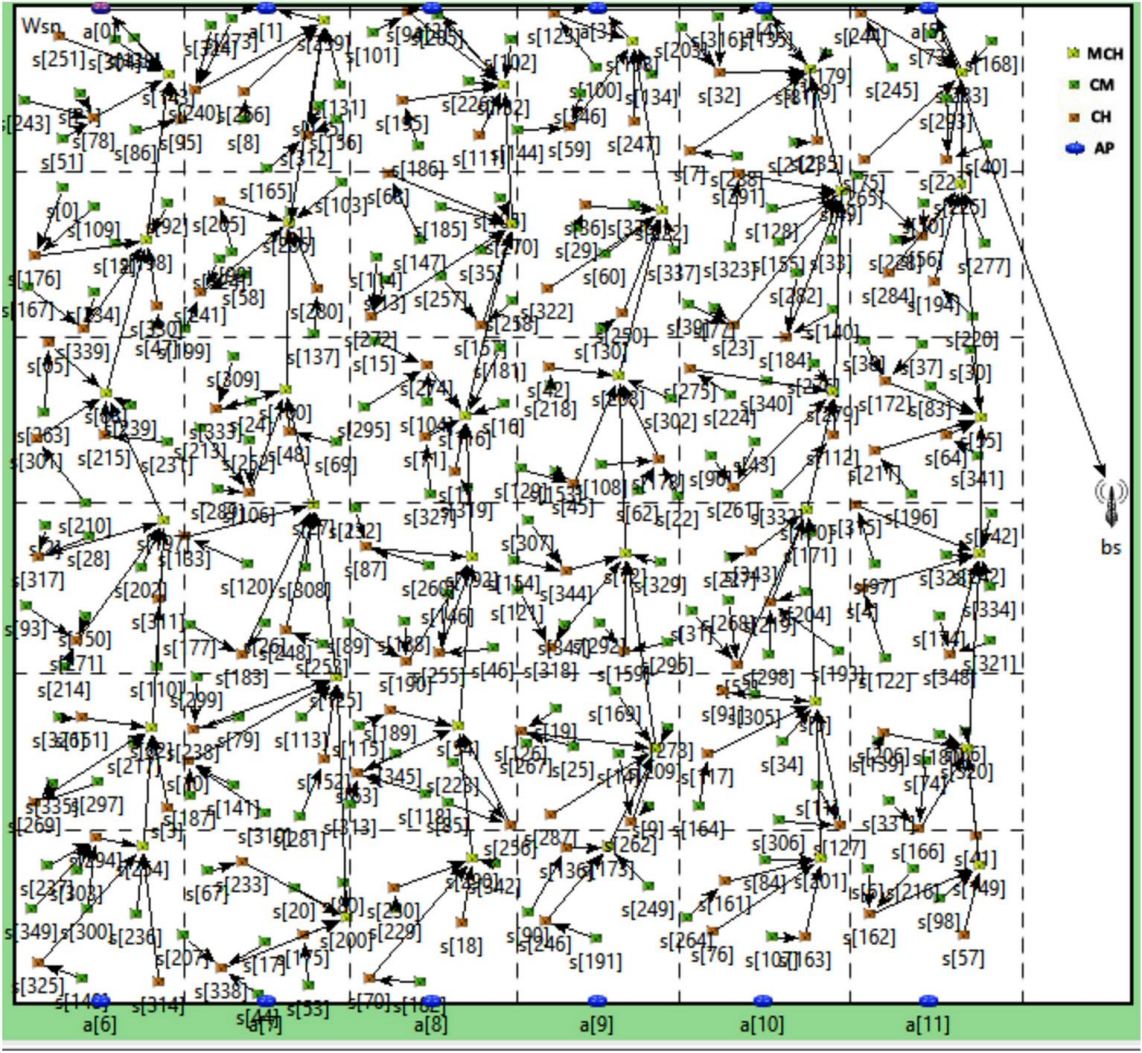
Data transmission path between CMs, CHs, MCHs, Data mule and the BS.

For comparison point of view, the size and number of clusters are taken considering the scenario proposed in FBECS ^20^. The size of a mega-cluster is taken as 50 X 50 ${m}^{2}$ and thus size of each cluster is 25 X 25 ${m}^{2}$. The network is divided into 6 layers and 36 mega-clusters. Figure 8 shows real deployment scenario of nodes on OMNeT platform and data transmission route for CMs, CHs, MCHs and data mules to the BS.

The position of the anchor points ${AP}_{0 },{AP}_{1 },{AP}_{2 },{AP}_{3 },{AP}_{4 }and {AP}_{5}$ are (0, 25), (0, 75), (0, 125), (0, 175), (0, 225) and (0, 275) respectively. Data mule 1 moves in the path joining these points when the round number in odd. The position of the anchor points ${AP}_{6 },{AP}_{7 },{AP}_{8 },{AP}_{9 },{AP}_{410 }and {AP}_{11}$ are (300, 25), (300, 75), (300, 125), (300, 175), (300, 225) and (300, 275) respectively. Data mule 2 moves in the path joining these points when the round number in even.

### Comparison of Node Failures: First, Last and Half of Network Nodes

**Fig. 9:**
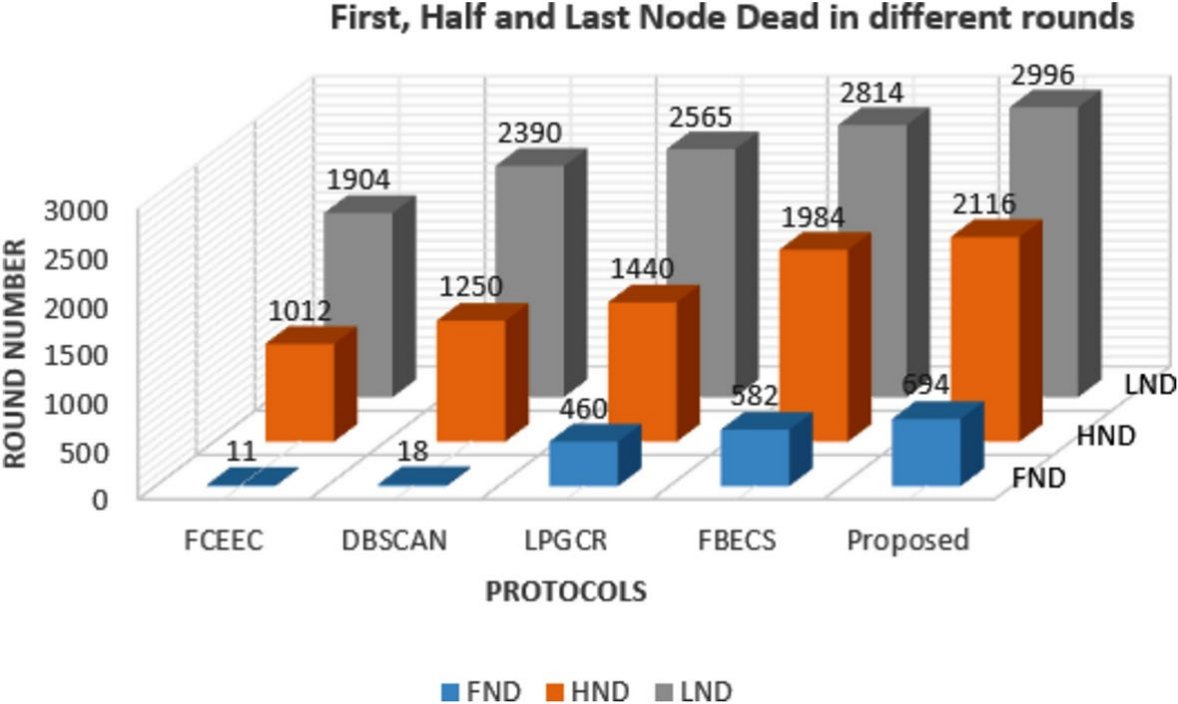
FND, HND and LND of the network for 600 sensors.

**Fig. 10:**
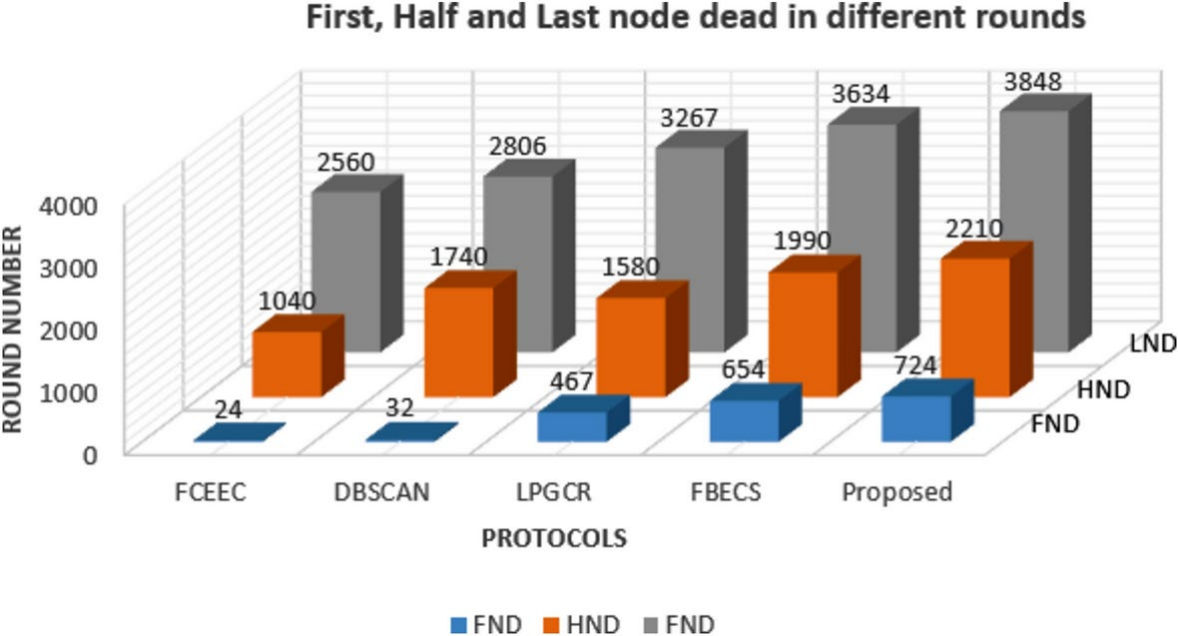
FND, HND and LND of the network for 1200 sensors.

Once the network has been set up, the main aim is to collect maximum data from the specified area considering life of a node on top priority. This is because the failure of any node can result in certain parts of the target area being left uncovered which ultimately leads to a degradation in the overall performance of the system in meeting its requirements. Figures 9 and 10 represents the graph plotted between corresponding number of rounds for different protocols after which first node, last node and half number of nodes are dead for the deployment scenarios involving of 600 and 1200 sensors respectively. We have compared the outcomes of proposed protocol with existing protocols FCEEC, DBSCAN, LPGCR and FBECS in terms of first node dead (FND), half node dead (HND) and last node dead (LND).

The result obtained for a deployment of 600 nodes in the network as shown in Figure 9. The proposed protocol improves network life (i.e., LND) by 36%, 20%, 14% and 6% as compared to FCEEC, DBSCAN, LPGCR and FBECS respectively. Also, HND is prolonged by 52%, 41%, 32% and 6% with respect to FCEEC, DBSCAN, LPGCR and FBECS respectively. The first node is dead after 11 rounds, 18 rounds, 460 rounds, 582 rounds and 694 rounds in FCEEC, DBSCAN, LPGCR, FBECS and proposed one respectively.

The result obtained for a deployment of 1200 nodes in the network as shown in Figure 10. It improves network life by 33%, 27%, 15% and 5% in contrast to FCEEC, DBSCAN, LPGCR and FBECS respectively. Also, HND is prolonged by 53%, 21%, 28% and 10% as compared to FCEEC, DBSCAN, LPGCR and FBECS respectively. The first node is dead after 24 rounds, 32 rounds, 447 rounds, 654 rounds and 724 rounds in FCEEC, DBSCAN, LPGCR, FBECS and proposed one respectively.

It can be inferred from both the graph that the proposed scheme performs better than existing protocols in both the scenarios.

### Comparison in terms of Dead Nodes

**Fig. 11:**
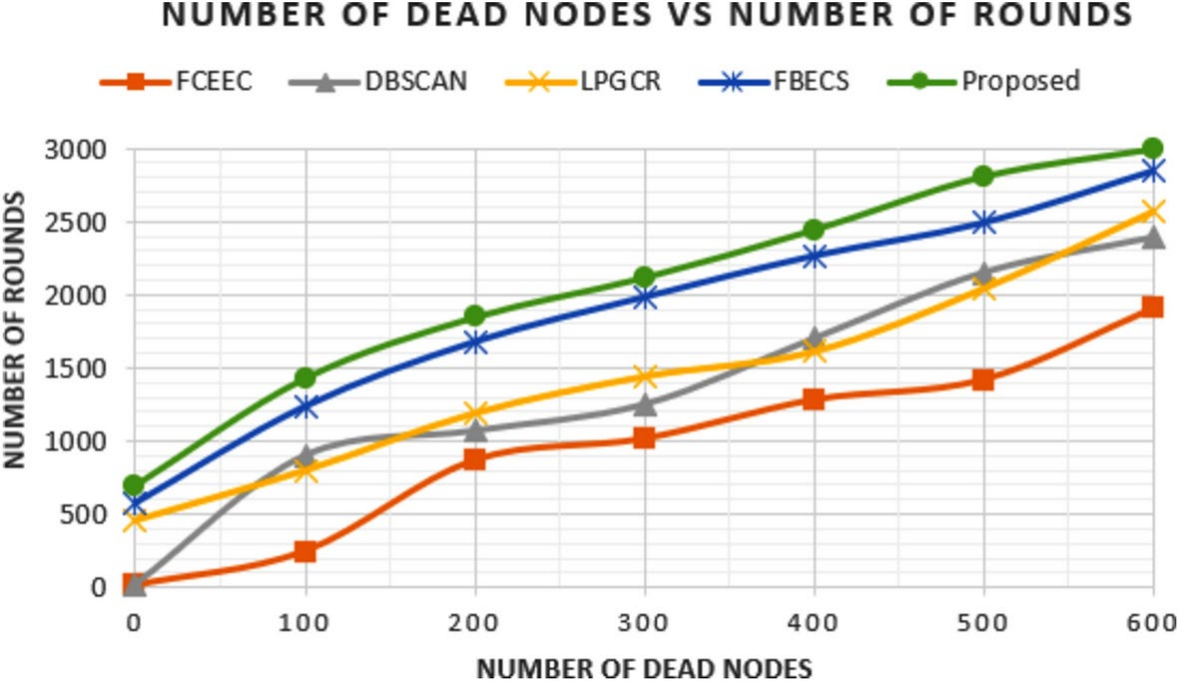
Graph plotted between number of dead nodes in respective round number for 600 sensors.

**Fig. 12:**
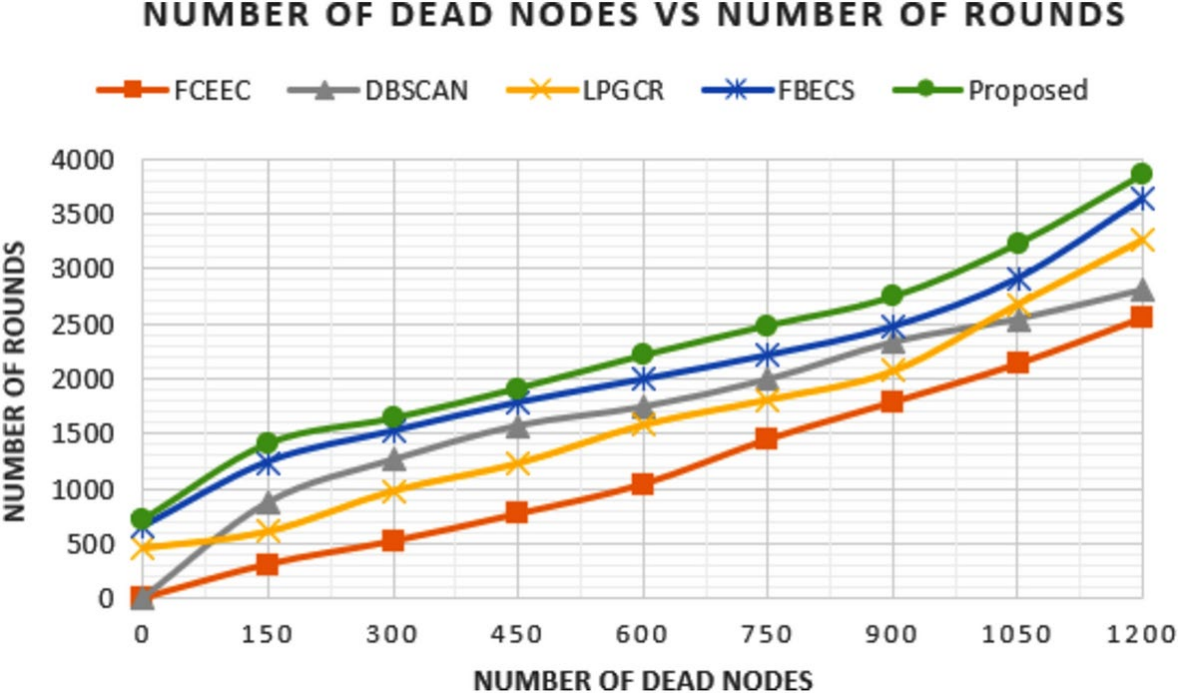
Graph plotted between number of dead nodes in respective round number for 1200 sensors.

The amount of information collected from the network and coverage area of this information depends on the number of alive nodes in the network. Figures 11 and 12 represents the graph plotted between number of round and corresponding number of dead nodes for the deployment scenarios involving of 600 and 1200 sensors respectively. We have compared the outcomes of proposed protocol with existing protocols FCEEC, DBSCAN, LPGCR and FBECS. For the first deployment scenario of 600 nodes in the network, all the nodes are dead after 1904 rounds, 2390 rounds, 2565 rounds, 2844 rounds and 2996 rounds for FCEEC, DBSCAN, LPGCR, FBECS and proposed protocol respectively. For the second deployment scenario of 1200 nodes in the network, all the nodes are dead after 2560 rounds, 2806 rounds, 3267 rounds, 3634 rounds and 3848 rounds for FCEEC, DBSCAN, LPGCR, FBECS and proposed protocol respectively. It can be inferred from the graph that the number of dead nodes is considerably less in the proposed protocol when compared with that of existing protocols. The prolonged lifespan of nodes can be attributed to the cluster size selection which is based on the threshold transmission distance and residual energy as a parameter for CH selection. Centralized nature of the protocol is also a key reason for less energy exhaustion of the sensors as it has minimal role in overhead tasks such as cluster formation, CH selection etc.

### Comparison in terms of Residual Energy

**Fig. 13:**
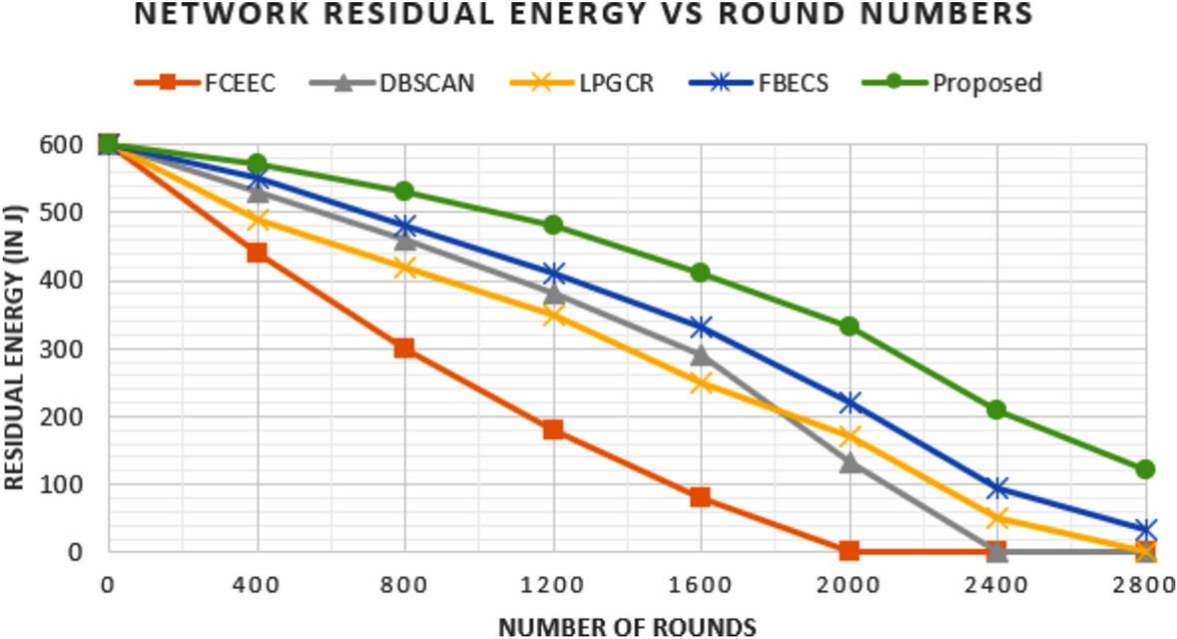
Graph plotted between network residual energy nodes in respective round number for 600 sensors.

**Fig. 14:**
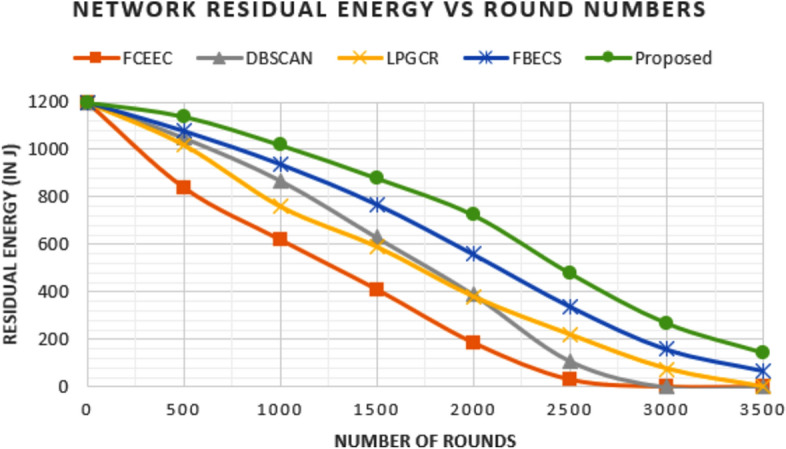
Graph plotted between network residual energy nodes in respective round number for 1200 sensors.

The energy of the network decreases with progress of rounds as sensor nodes drain energy in performing assigned tasks. Figures 13 and 14 show the graph plotted between number of rounds and residual energy of the network for the deployment of 600 nodes and 1200 nodes respectively.

For deployment of 600 nodes, network deplete 50% and 75% of its energy after 804 rounds, 1582 rounds, 1401 rounds, 1723 rounds & 2110 rounds and 1370 rounds, 1980 rounds, 2090 rounds, 2240 rounds & 2710 rounds for FCEEC, DBSCAN, LPGCR, FBECS and proposed protocol respectively. Similarly, for deployment of 1200 nodes, network deplete 50% and 75% of its energy after 1740 rounds, 2210 rounds, 2260 rounds, 1910 rounds & 2256 rounds and 1370 rounds, 1980 rounds, 2090 rounds, 2614 rounds & 2904 rounds for FCEEC, DBSCAN, LPGCR, FBECS and proposed protocol respectively.

The comparison evidently shows that the proposed protocol maintains a much higher level of residual energy in the network compared to existing protocols, across both deployment scenarios. As a result, the network lifespan is significantly extended.

### Time Complexity

The proposed protocol is centralized and BS driven protocol designed for network having n number of homogeneous nodes. The BS collects information about the network such as node locations, energy levels, etc. The worst-case time complexity of our routing scheme for network topology discovery is O(n). In the proposed protocol, clustres are formed once based on proximity of the sensor nodes. As BS evaluates every node for cluster membership, the complexity of cluster formation is O(n). As BS evaluate nodes for selecting CH based on multiple criteria such as energy level, distance from centroid etc., the time complexity for CH selection is O(n log n). As BS computes routes for CMs to CHs, CHs to MCHs, MCHs to MCHs and MCHs to data mules, the time complexity for routing path computation is O(e + n log n) where e is the number of edges. As BS creates schedules for each node transmissions to avoid collision, the time complexity for generating a schedule for n nodes is O(n).

The total time complexity of the proposed centralized routing algorithm is typically O(n log n + e) for cluster formation, CH selection, MCH selection and routing within and between clusters.

The paper presents a novel routing protocol designed to address the limitations of existing protocols by focusing on energy conservation for sensor nodes. By introducing new network divisions and data transmission schemes, the protocol outperforms existing ones like FCEEC, DBSCAN, LPGCR, and FBECS in terms of network lifespan, residual energy and overall performance. These improvements are achieved by minimizing energy consumption for major network tasks such as network formation, CH and MCH selection & data flow route discovery. All of these tasks are handled by the BS instead of deployed nodes. The transmission distance of nodes is limited to the threshold range defined by the sensor node’s radio energy model, ensuring significant energy savings.

The use of an odd–even data flow strategy ensures balanced traffic and energy consumption, effectively eliminating the hotspot problem and reducing the likelihood of network failure. Most of the energy of sensor nodes is dedicated to primary activities such as sensing and transmitting data to the BS, rather than overhead tasks. These combined factors significantly enhance energy efficiency, leading to longer network lifespan and greater stability. By incorporating various energy-saving strategies into a single routing protocol, the proposed protocol demonstrates superior performance compared to existing protocols in terms of both network longevity and stability, with mathematical analysis confirming its positive impact on the overall network lifespan.

## Conclusion and Future Works

The proposed EEMCR (Centralized Mega-Cluster-Based Routing Protocol) offers superior performance due to several key factors. As a centralized protocol, it minimizes the involvement of sensor nodes in cluster formation, CH selection and route discovery which significantly reducing energy consumption in overhead tasks. The roles of CHs and MCHs are rotated systematically, ensuring balanced energy consumption across the network. Additionally, data flow is optimized based on odd–even round numbers enhancing network efficiency and uniform distribution of data traffic. This approach reduces the likelihood of the hotspot problem, leading to a longer network lifespan.

The protocol ensures that no sensor node exceeds the threshold transmission distance defined by the radio energy model, minimizing energy depletion. This ensures energy depletion of sensor node at rate of ${d}^{2}$ which is remarkably very less than ${d}^{4}$. Data aggregation occurs at two levels reducing the volume of data that needs to be transmitted and conserving energy in relay nodes. The inclusion of data mules further relieves boundary nodes from the burden of transmitting large data over long distances. These combined strategies significantly extend the energy life of sensor nodes enhancing the overall stability and lifespan of the network.

The proposed protocol eliminates the limitations of existing protocols like FCEEC, DBSCAN, LPGCR, and FBECS by addressing key challenges such as hotspot issues, long distance transmissions and overhead energy loss of nodes in clustering. It incorporates data mules to further enhance network longevity ensuring more efficient energy use and extending the overall lifespan of the network. Simulation results show that EEMCR outperforms existing protocols such as FCEEC, DBSCAN, LPGCR, and FBECS in terms of key performance metrics including the number of dead nodes, average energy depletion and overall network lifetime.

In future, reduction in the data delivery time and CH rotation frequency can be considered for optimization of the protocol.

## Data Availability

All data generated or analyzed during this study are included in this published article.
